# Experimental in-vitro investigation on Epi-Off-Crosslinking on porcine corneas

**DOI:** 10.1371/journal.pone.0249949

**Published:** 2021-04-15

**Authors:** Federica Boschetti, Debora Conti, Elvira M. Soriano, Cosimo Mazzotta, Anna Pandolfi

**Affiliations:** 1 Chemistry, Materials, and Chemical Engineering Department, Politecnico di Milano, Milan, Italy; 2 Department of Medicine, Surgery and Neurosciences, Post Graduate Ophthalmology School, University of Siena, Siena, Italy; 3 Siena Crosslinking Center, Siena, Italy; 4 Civil and Environmental Engineering Department, Politecnico di Milano, Milan, Italy; Save Sight Institute, AUSTRALIA

## Abstract

**Aim:**

To evaluate quantitatively the effects of the Epi-Off-CXL irradiance dose on the stromal stiffening of pig corneas.

**Setting:**

Laboratory of Biological structures (LaBS), Politecnico di Milano, Milano, Italy.

**Methods:**

Inflation tests have been carried on 90 excised and de-epithelized pig corneas, monitoring the change of configuration of the corneal dome at specific pressures. Test have been carried out twice on each cornea, once before and once after Epi-Off-CXL performed at a constant irradiance of 9 mW/cm^2^ and variable UV-A exposure times. Corneas were grouped according to the exposure time (2.5, 5, 10, 15 and 20 min), proportional to the irradiation dose (1.35, 2.7, 5.4, 8.1, and 10.8 J/cm^2^). A theoretical model based on linearized shell theory has been used to estimate the increment of the corneal stiffness.

**Results:**

The linearized shell theory allowed to establish a quantitative relation between the increment of the stiffness parameters and the irradiation dose. Relative to the pre-treatment values, in all experiments the post-treatment corneal stiffness revealed a pronounced increase. In general, the stiffness gain increased with the exposure time. No significant differences in stiffening was observed between tests conducted at 2.5, 5, and 10 min exposure.

**Conclusions:**

Qualitatively, the effectiveness of accelerated CXL treatments observed in pig corneas complies very well with in-vivo clinical results in humans, suggesting that experimental data in pigs can be very useful for the design of the procedure in humans. A larger irradiation dose provides a larger increment of the corneal stiffness. Due to the biological variability of the tissues, however, it is difficult to distinguish quantitatively the level of the reinforcement induced by accelerated protocols (low doses with < = 10 min exposure), less prone to induce damage in the corneal tissue. Therefore, the definition of personalized treatments must be related to the actual biomechanics of the cornea.

## 1. Introduction

The cornea, the outermost tissue of the eye, provides mechanical protection to the inner parts of the eye. The cornea has an average thickness of 570 μm in humans [1] and 975 μm in pigs [2], and is composed by aP main layers: the anterior epithelium, the Bowman lamina, the thick carrying structure called stroma, the Descemet lamina and the posterior endothelium [3].

The stroma is composed by a matrix of elastin and proteoglycans embedding collagen fibrils, organized into a hierarchical architecture able to provide the necessary mechanical stiffness [4]. The mechanical behavior of the cornea depends on several geometrical and mechanical factors, such as topography, thickness, composition, microstructure, and mechanical properties of the tissue [5]. The spherical shape of the cornea, structurally a thin shell, is achieved by the response of the tissue to the action of the intraocular pressure (IOP) due to the internal fluids [6].

Alterations of the regular curvature of the cornea lead to poor vision and various optical aberrations. Typical examples are ectasia and keratoconus. Keratoconus is a genetic progressive non inflammatory disease of the cornea distinguished by paracentral corneal thinning, decrease of crosslinks between collagen fibrils, and evident reduction of mechanical stiffness [7]. Keratoconus corneas are very compliant and lose the spherical shape in favor of a conical shape, impairing the focusing abilities of the eye.

The prime treatment of keratoectasia consists of an early intervention to halt or slow down the progression of the pathology. Several options are available in clinical practice, including conservative approaches, that preserve the corneal integrity, and more invasive approaches, that imply the partial or total removal of the cornea [7]. A treatment of keratoconus in its earlier stages might avoid corneal transplant.

Among conservative treatments, the therapeutic collagen crosslinking (CXL) technique stands as the sole procedure able to improve the mechanical properties of the cornea and to prevent keratectomy [8].

Corneal CXL has surged as one of the standard treatments for keratoconus. CXL (Dresden protocol) consists of a collagen photo-polymerization reaction which is activated through the administration of riboflavin (B2 vitamin) on the cornea, followed by 30 min UV-A irradiation at a wavelength of 370 nm and 3mW/cm^2^ irradiance [8]. The chemical reaction produces oxygen radicals and in turn, via photo-polymerization, creates crosslinks between the collagen fibrils located in the anterior stroma. As effect of crosslink creation, collagen fibrils thicken and stiffen, making the cornea less prone to further shape changes [5, 8].

The clinical practice has been using several CXL protocols, differing one from the other for duration and intensity of the UV-A irradiation, for amount and timing supply of riboflavin, and for direct or indirect exposure of the stroma. The epithelium represents a barrier to the spreading of riboflavin and implies a larger oxygen consumption [9], resulting in a reduced stiffening of the tissue [10, 11]. The so called Epi-Off-CXL procedures are carried out directly on the stroma, after the removal of the epithelium [12], and are considered among the most effective. Further studies demonstrated that both too short or too prolonged irradiations do not improve the outcomes of the CXL treatment [13], and that the total amount of irradiated energy (or irradiance dose) remains the most significant parameter in terms of stiffening effects [14].

An open question is the quantification of the efficacy of the treatment in mechanical terms, and the definition of personalized protocols to be used to attain the desired corneal reinforcement. A recent experimental study on rabbit corneas pointed out that the CXL supplied irradiance dose and the attained overall corneal stiffening are not linearly correlated [15]. Furthermore, the quantitative evaluation of therapeutic CXL requires further investigations, since the treatment effects reduce noticeably across the corneal thickness, thus the in-depth increase of mechanical stiffness is not uniform, and the treatment efficiency reduces in time, due to the progressive saturation of the free reactive collagen residues involved in the reaction [16].

The present study describes an experimental Epi-Off-CXL campaign carried out on porcine corneas with the goal to evaluate quantitatively the effects of the irradiance dose on the stromal stiffening. Experiments are interpreted with a linearized theory of elastic shells to estimate the equivalent increment of the corneal stiffness.

The research aims at providing quantitative indications on the dose necessary to achieve the desired corneal stiffness without inducing a permanent damage, due to prolonged exposure of the tissue to the irradiation. The availability of quantitative data in terms of stiffening versus irradiation dose can be taken as a starting point for the definition of an optimal dose in patient specific applications.

## 2. Materials and methods

A large sample consisting of 90 porcine eyes was acquired, in sixteen distinct deliveries, at a local abattoir (Fumagalli Industria Alimentari S.p.A., Via Briantea, 18, 22038 Tavernerio CO) from pigs aged 9 months within an hour post mortem. Pig eyes were supplied with eyelid, cheek, and forehead tissues and immediately stored in the refrigerators of the LaBS, in order to preserve the mechanical and optical integrity of the corneas for two days. No macroscopic changes of the eye were observed within two days. All specimens, de-epithelized and including an annulus of scleral tissue, underwent inflation tests at room temperature, before and after a CXL treatment. CXL was performed at different irradiation doses (1.35, 2.7, 5.4, 8. and 10.8 J/cm^2^) by using a constant irradiance of 9 mW/cm^2^. The 9 mW/cm^2^ irradiance was chosen because it is currently used with success in accelerated CXL protocols (A-CXL), providing visual outcomes, in terms of keratoconus stabilization, comparable with the ones obtained with the conventional Dresden protocol [17, 18]. The 9 mW/cm^2^ irradiance is suitable for a standardized conservative treatment of all thickness primary and iatrogenic ectatic corneas, without inducing tissue damage [19].

**Fig 1:**
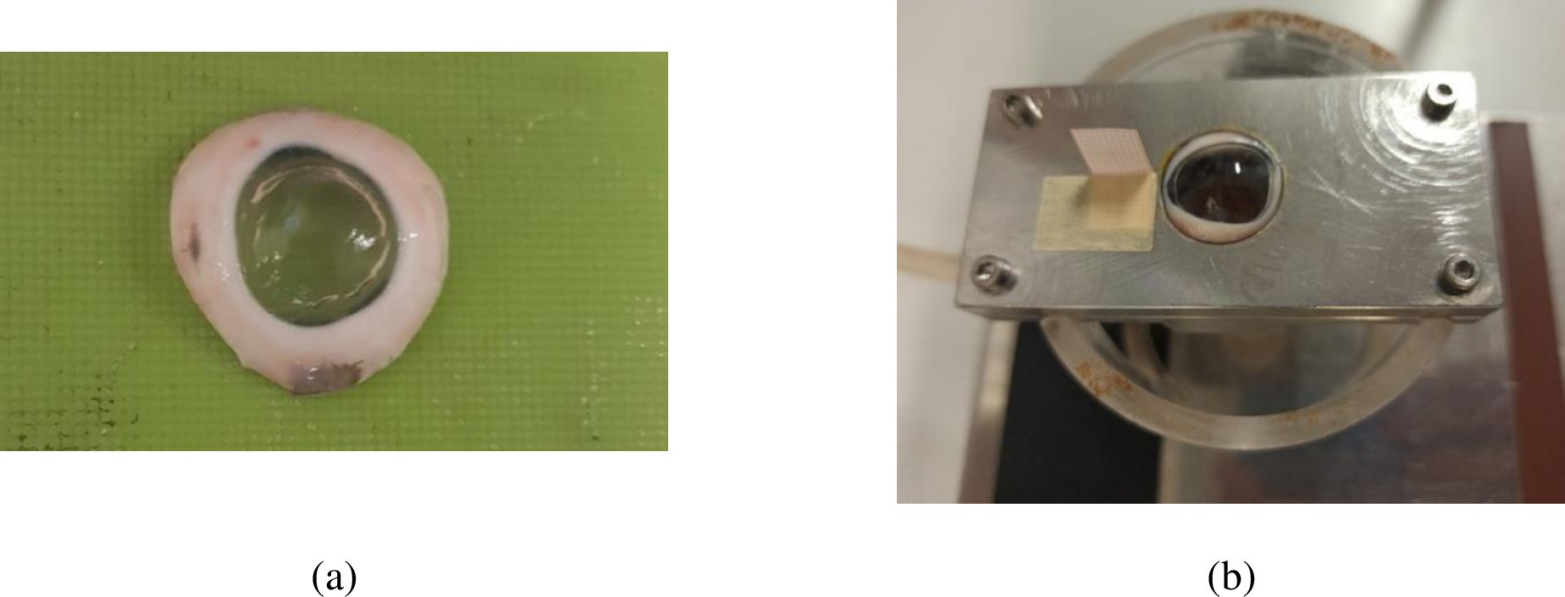
(a) Cornea specimen de-epithelized, incised, and cleaned for the inflation tests. (b) Grip system used for clamping the cornea specimens.

An accurate protocol was defined to avoid edema of the corneas. Tests were performed within one hour of the dissection and, during the preparation of the samples, tissues were wetted with a physiological solution. After the disposal of cheek, forehead and eyelid, the epithelium was removed from the intact eyeball with a scalpel. The posterior eye portion was eliminated from the globe with an all-round equatorial incision performed with a scalpel. Next, the not relevant tissues (choroid, lens, iris) of the anterior eye were disposed, leaving an annular strip of scleral tissue for clamping, Fig 1A.

To avoid slipping, specimens were clamped at the sclera in a grip consisting of two rectangular plates. The bottom plate is equipped with a spherical cup (24 in-plane diameter) where the cornea sample was positioned. The spherical cup has a central 18 mm diameter circular hole. The top plate has a central circular 20 mm diameter hole concentric with the bottom one, Fig 1B. The spherical cap surface was slightly roughened to facilitate the positioning of the samples and it was surrounded by an O-ring to avoid leakage. The two plates were assembled with four tightening screws, located in proximity of the vertices, to exclude the onset of stress concentrations or unnatural pre-tensioning.

**Fig 2:**
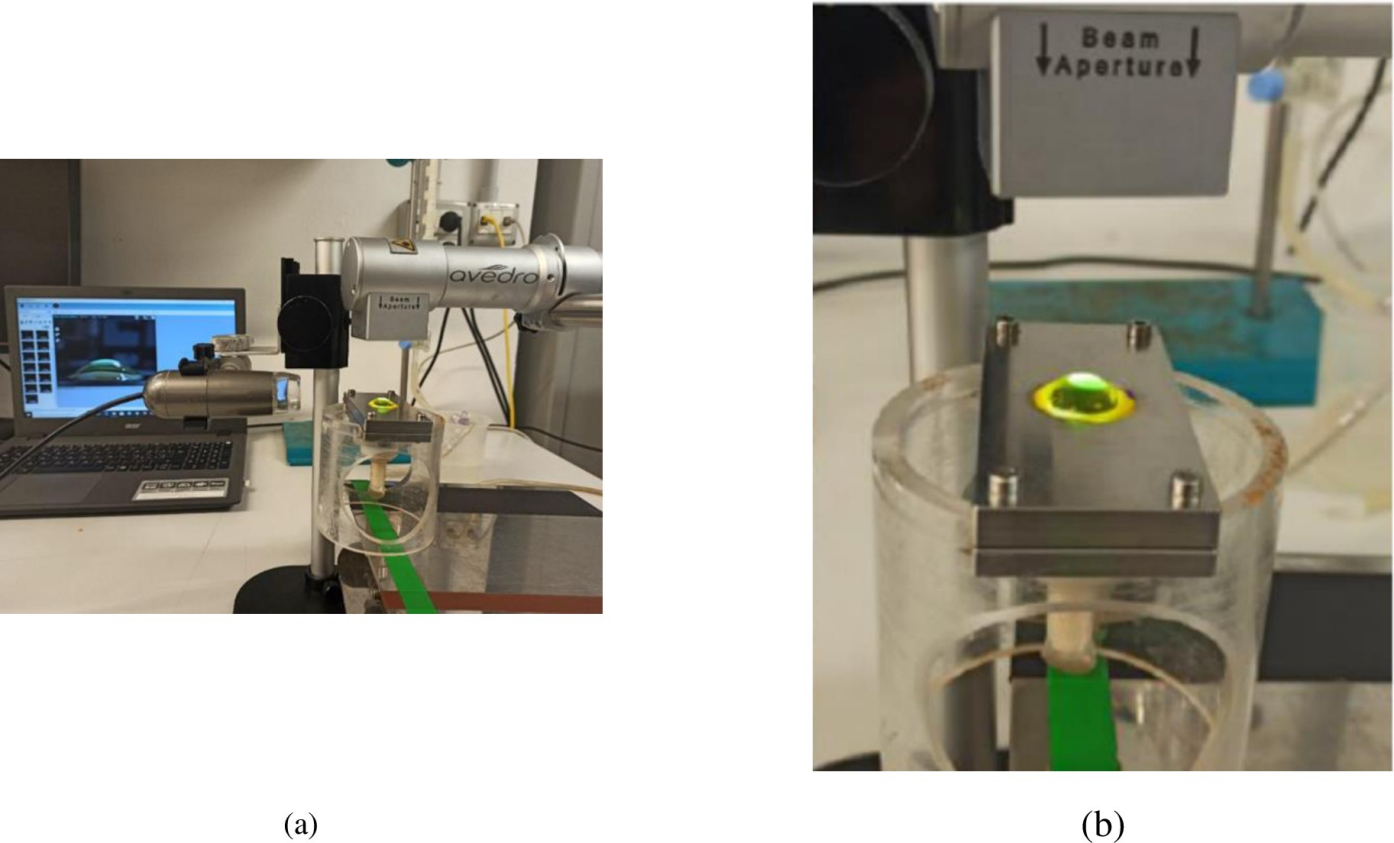
(a) Experimental setup. From left to right: personal computer for image acquisition, Pro Lite Dino Camera; clamping system where the cornea sample is mounted; water column; piping system; constant emission UV-A lamp. (b) AVEDRO lamp used for the UV-A activation of riboflavin.

During the inflation tests, cornea specimens were subjected to a posterior pressure (1.8 to 30 mmHg in steps of 2.5 mmHg) induced by a column of NaCl solution to simulate the effect of a growing intraocular pressure. Pressure was directly applied with an open pipe circuit. Before conducting the monitored inflation tests, each specimen was subjected to three loading-unloading pressure cycles in the range 1.8 to 30 mmHg. Images including the entire profiles of the anterior surface of the cornea were acquired at regular intervals by means of a Pro Lite Dino Camera and stored on a personal computer. Images were later analyzed with the software ImageJ to track the anterior cornea displacements as a function of the pressure. The experimental setup used during the execution of the experimental tests is shown in Fig 2A.

The CXL procedure was conducted by soaking the cornea with dextran-free plus hydroxyl-propyl-methyl-cellulose (HPMC) 0.1% riboflavin isotonic solution for 10 minutes, dosing 2 drops every 3 minutes, see Fig 1B. Riboflavin was activated by UV-A irradiation using an AVEDRO’s lamp, Fig 2B, able to deliver a constant irradiance of 9mW/cm^2^ at 370 nm wavelength over a circular area of 7.5 mm diameter. During the irradiation, one drop of riboflavin was dosed every 2.5 minutes.

Twenty-two corneas were treated with the A-CXL, consisting in 10 minutes of riboflavin soaking followed by 10 minutes of exposure to UV-A, using an irradiance of 9mW/cm^2^, for total energy dose 5.4 J/cm^2^. The other corneas were divided in four groups and underwent CXL with different exposure times (and different total energy doses). Specifically, seventeen corneas received 2.5 min UV-A exposure (1.35 J/cm^2^), sixteen 5 min (2.7 J/cm^2^), eighteen 15 min (8.1 J/cm^2^) and seventeen 20 min (10.8 J/cm^2^).

After the CXL treatment, corneas were sectioned in strips to measure the average thickness, that was used in the subsequent numerical studies.

Corneal displacements measured during the test were elaborated by means of the linearized shell theory to obtain estimates on the average mechanical response of the cornea. The linearized shell theory is based on the assumptions of a perfectly spherical shell cap, subtended by an angle 2*α*, of uniform thickness *t*, mid-surface radius *R*, in-plane radius *S* = *D*/2, constrained at the boundary with pins, obeying a linear elastic isotropic material law (Hooke) with elastic modulus *E* and Poisson’s coefficient *v* (see [20] for a synthetic description of the theory). The expressions used to compute the stress and the strain in the meridian (elevation angle *φ*) and in the circumferential (azimuth angle *θ*) directions of the shell under a pressure *p* acting on the posterior surface are:

$$\sigma_{\varphi }=\frac{N_{\varphi }}{t},\;\sigma_{\theta }=\frac{N_{\theta }}{t}$$ (1)

$$\varepsilon_{\varphi }=\frac{N_{\varphi }-vN_{\theta }}{Et},\;\varepsilon_{\theta }=\frac{N_{\theta }-vN_{\varphi }}{Et}$$ (2)

where the membrane stresses (forces per unit of length) are defined as

$$N_{\varphi }=\frac{pR}{2},\;N_{\theta }=\frac{pR}{2}[1-(1-v)e^{-\lambda \psi }cos\;cos\;\lambda \psi ]$$

and the constant *λ* and the angle *ψ* are:

$$\lambda ={\left[3(1-v^{2})\right]}^{1/4}\frac{R^{1/2}}{t^{1/2}},\;\psi =\frac{S}{R}-\varphi .$$

The apex displacement *w* measured experimentally at physiological IOP was used to estimate the average secant elastic modulus *E* (assumed to be valid for all the cornea) as:

$$E=\frac{pR^{2}}{w\;2t}(1-v)(1-e^{-\lambda \psi }\;cos\;cos\;\lambda \psi )$$ (3)

Eqs (1) and (2) were used to construct idealized stress-strain curves for the cornea material before CXL and Eq (3) was used to estimate the average elastic modulus of the tissue at the physiological IOP, assumed to be 15 mmHg, before and after CXL. The pre-CXL average and uniform elastic modulus at the physiological IOP is denoted with *E*_*b*_. The post-CXL average and uniform equivalent elastic modulus at the physiological IOP, computed for each group of corneas, is denoted with *E*_*a*_. Note that Eq (3) is not properly valid for treated corneas, characterized by inhomogeneity across the thickness (stiffer in the anterior stroma, softer in the posterior stroma), and it must be seen only as an approximated qualitative estimate. Stress versus strain curves for treated corneas are not reported, because of the impossibility to distinguish the stress in the anterior and in the posterior stroma.

In order to apply the shell theory, geometrical measurements corresponding to the unstressed configuration were taken on each cornea at the minimum value of pressure applied in the experiments (1.839 mmHg). The parameters necessary for the construction of the unstressed cornea are: the average in-plane diameter *D* = 2*S* (considering both nasal-temporal NT and superior-inferior SI meridians), the average curvature *R* (considering both NT and SI meridians), the thickness at the apex, and the elevation *H* of the apex. Measures were taken on the anterior surface, computed on the posterior surface by using a simple geometric construction described in [20] and averaged to the mid surface of the cornea before the application to the shell theory.

**Fig 3:**
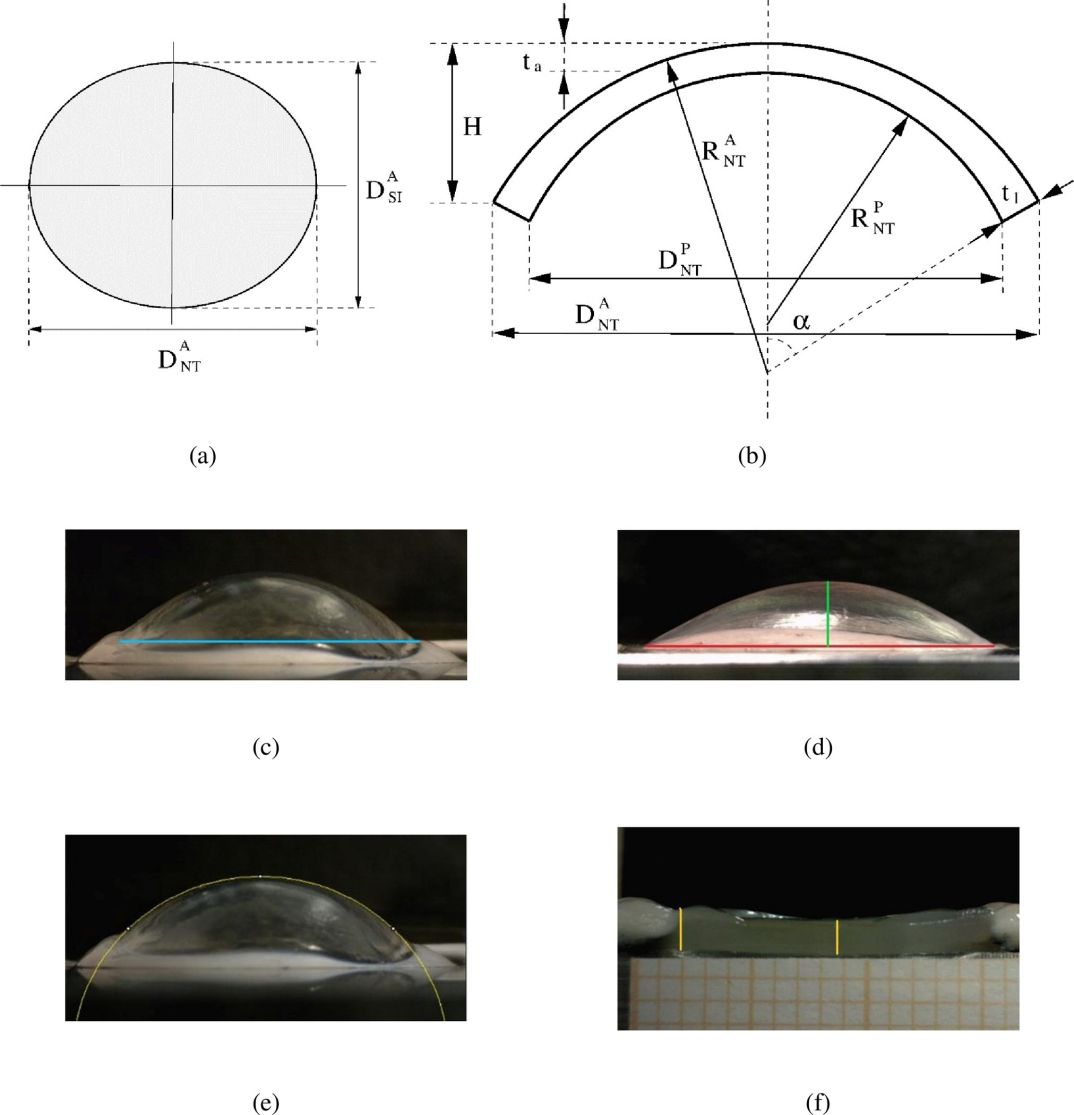
(a) In-plane view of the elliptical shape of the pig cornea. (b) NT corneal section: geometrical meaning of the parameters acquired through the imaging software: in-plane anterior diameter $D_{NT}^{A}$; in-plane posterior diameter $D_{NT}^{P}$; anterior curvature *R*; posterior curvature $R_{NT}^{P}$; apex elevation H; limbus thickness, *t*_*l*_; apex thickness *t*_*a*_. (c-f) Acquisition of the main geometrical parameters of the cornea through imaging software. (c) SI in-plane anterior diameter $D_{SI}^{A}$ (blue line). (d) NT in-plane anterior diameter $D_{NT}^{A}$ (red line) and apex elevation H (green line). (e) Anterior curvature $R_{NT}^{A}$. (f) Limbus thickness, *t*_*l*_, (yellow line on the left) and apex thickness *t*_*a*_ (yellow line at the center).

Fig 3A shows schematically the in-plane projection of the pig cornea, with different NT and SI diameters, while Fig 3B illustrates, in the NT meridian section, the meaning of the design parameters for the cornea model. Fig 3C–3F show how the measurements were obtained from the images: the in-plane diameter in the SI direction, Fig 3C; the elevation and the in-plane diameter in the NT direction, Fig 3D; the anterior NT curvature, Fig 3E; limbus and apex thicknesses, Fig 3F. The average values collected in the experiments are listed in Table 1.

**Table 1:** Geometrical parameters of the pig corneas.

Parameter	mm
$D_{NT}^{A}$	17.996 ± 1.160
$D_{NT}^{P}$	16.571 ± 1.2016
$R_{NT}^{A}$	11.827 ± 0.974
$R_{NT}^{P}$	10.412 ± 1.035
$D_{SI}^{A}$	14.852 ± 0.996
$D_{SI}^{P}$	13.436 ± 0.915
$R_{SI}^{A}$	9.926 ± 1.242
$R_{SI}^{P}$	8.459 ± 0.839
*H*	4.165 ± 1.652
*t*_*l*_	1.350 ± 0.363
*t*_*a*_	0.994 ± 0.198

Average and standard deviation values measured at 1.83 mmHg pressure on a partial sample of 47 specimens.

## 3. Results

**Fig 4:**
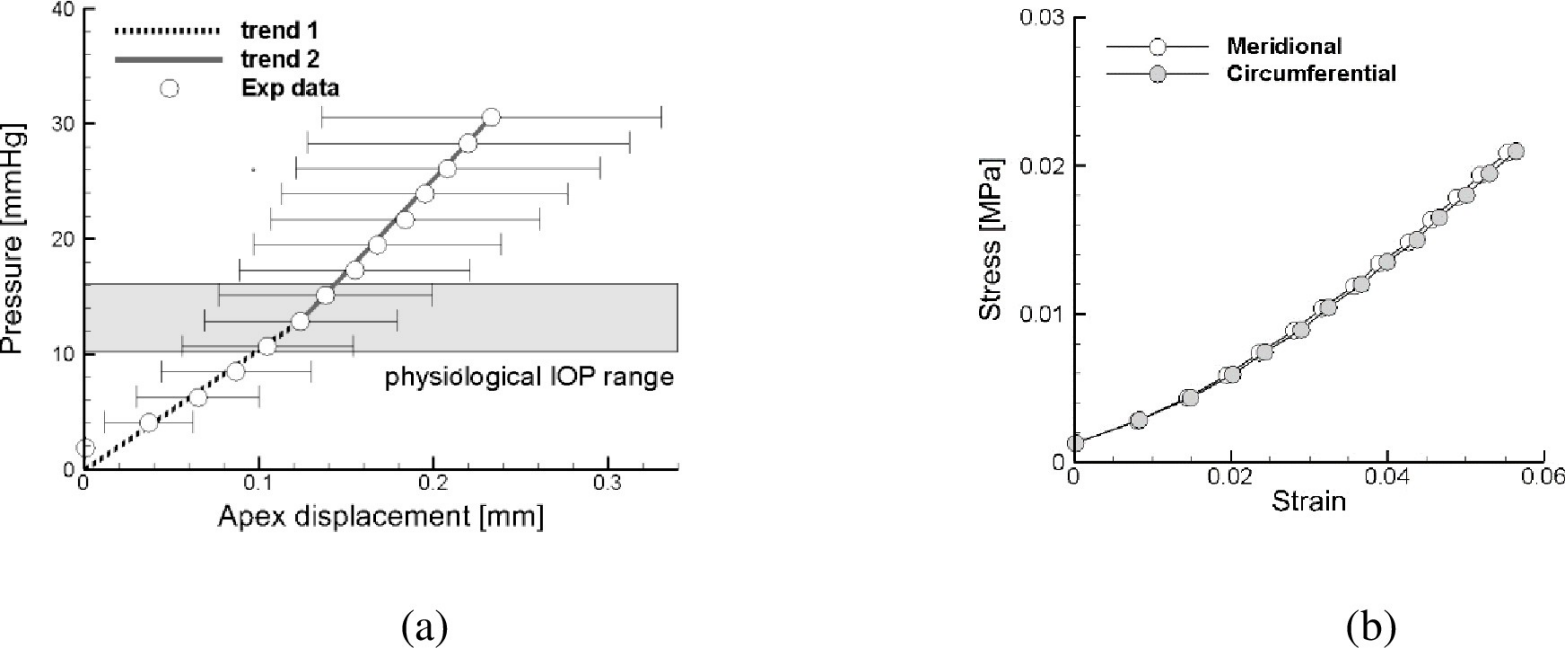
Inflation tests for untreated corneas. (a) Average values and standard deviation of the apex displacement versus pressure. (b) Biaxial stress-strain curves obtained from the shell theory using the average data.

Pressure versus apex displacement were obtained from the inflation tests conducted on untreated and treated corneas. Stress versus strain curves were obtained for untreated corneas. The results have been analyzed statistically, with reference to every irradiation protocol. Average and standard deviation values of the apex displacement as a function of the pressure for untreated corneas are collected in Table 2 (second column) and visualized in Fig 4A. In every specimen, the global IOP versus apex displacement plot shows a progressive increment in the corneal stiffness with a growing applied pressure, Fig 4A. The stiffness of the inflated cornea, which is the slope of the IOP-apex displacement curve, shows a marked increment around 10–16 mmHg IOP [21]. The biaxial stress-strain curves for untreated corneas obtained from the average data using the shell theory are visualized in Fig 4B. Values of stresses and strains are in the range of previous experimental results [20].

**Table 2:** Experimental inflation tests.

Pressure	Apex displacement [mm]					
[mmHg]	Untreated	2.5 min	5.0 min	10 min	15 min	20 min
		1.35 J/m^2^	2.7 J/m^2^	5.4 J/m^2^	8.1 J/m^2^	10.4 J/m^2^
1.839	0.001 ± 0.000	0.001 ± 0.000	0.001 ± 0.000	0.001 ± 0.000	0.001 ± 0.000	0.001 ± 0.000
4.045	0.037 ± 0.025	0.019 ± 0.015	0.019 ± 0.011	0.018 ± 0.014	0.014 ± 0.010	0.014 ± 0.008
6.252	0.065 ± 0.035	0.032 ± 0.019	0.032 ± 0.013	0.030 ± 0.016	0.026 ± 0.015	0.021 ± 0.010
8.459	0.087 ± 0.043	0.044 ± 0.018	0.046 ± 0.014	0.047 ± 0.019	0.036 ± 0.017	0.031 ± 0.009
10.665	0.105 ± 0.049	0.061 ± 0.018	0.059 ± 0.015	0.058 ± 0.025	0.043 ± 0.016	0.038 ± 0.011
12.872	0.124 ± 0.055	0.074 ± 0.019	0.070 ± 0.014	0.069 ± 0.026	0.053 ± 0.016	0.042 ± 0.008
15.078	0.138 ± 0.061	0.082 ± 0.020	0.081 ± 0.016	0.083 ± 0.029	0.062 ± 0.021	0.052 ± 0.011
17.285	0.155 ± 0.066	0.092 ± 0.019	0.089 ± 0.016	0.093 ± 0.034	0.069 ± 0.020	0.059 ± 0.008
19.492	0.168 ± 0.071	0.102 ± 0.018	0.097 ± 0.016	0.104 ± 0.034	0.079 ± 0.023	0.068 ± 0.009
21.698	0.184 ± 0.077	0.109 ± 0.018	0.105 ± 0.019	0.113 ± 0.033	0.086 ± 0.022	0.074 ± 0.012
23.905	0.195 ± 0.082	0.120 ± 0.019	0.114 ± 0.020	0.120 ± 0.031	0.092 ± 0.023	0.082 ± 0.012
26.112	0.208 ± 0.087	0.130 ± 0.018	0.123 ± 0.024	0.127 ± 0.034	0.102 ± 0.026	0.088 ± 0.012
28.318	0.220 ± 0.092	0.137 ± 0.020	0.132 ± 0.029	0.136 ± 0.034	0.107 ± 0.025	0.096 ± 0.014
30.525	0.233 ± 0.097	0.146 ± 0.022	0.137 ± 0.028	0.145 ± 0.036	0.117 ± 0.029	0.104 ± 0.012

Apex displacement as a function of the pressure for natural corneas and for CLX corneas with different UV-A exposure times and doses. Average values and standard deviation.

Average and standard deviation values of the apex displacement as a function of the pressure for treated corneas of different groups are also collected in Table 2 (third to seventh column).

**Fig 5:**
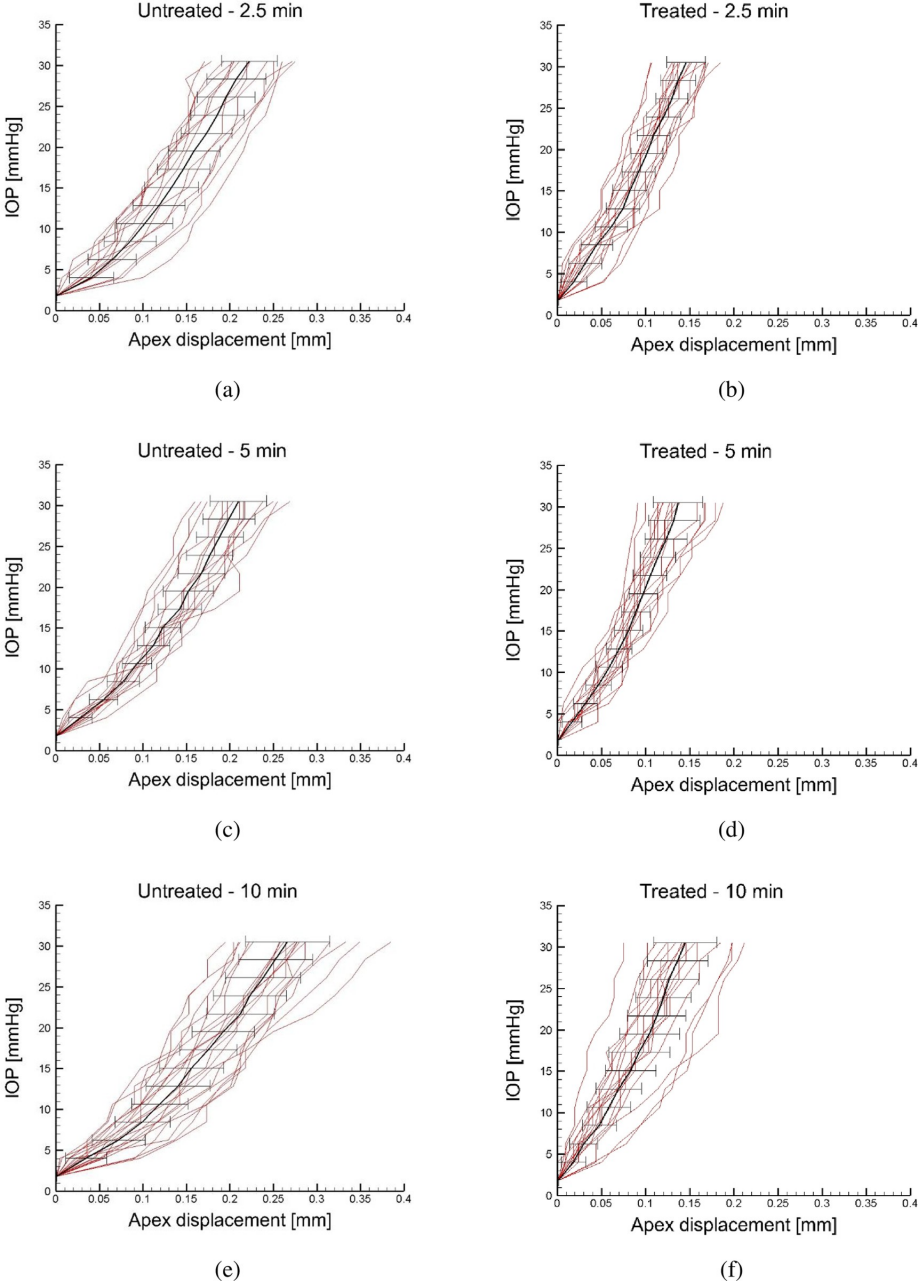
Inflation tests for the cornea groups irradiated for shorter times, 2.5, 5 and 10 minutes, respectively. IOP versus apex displacement: comparison between the untreated corneas and the treated corneas. Average values and error bars are also reported.

**Fig 6:**
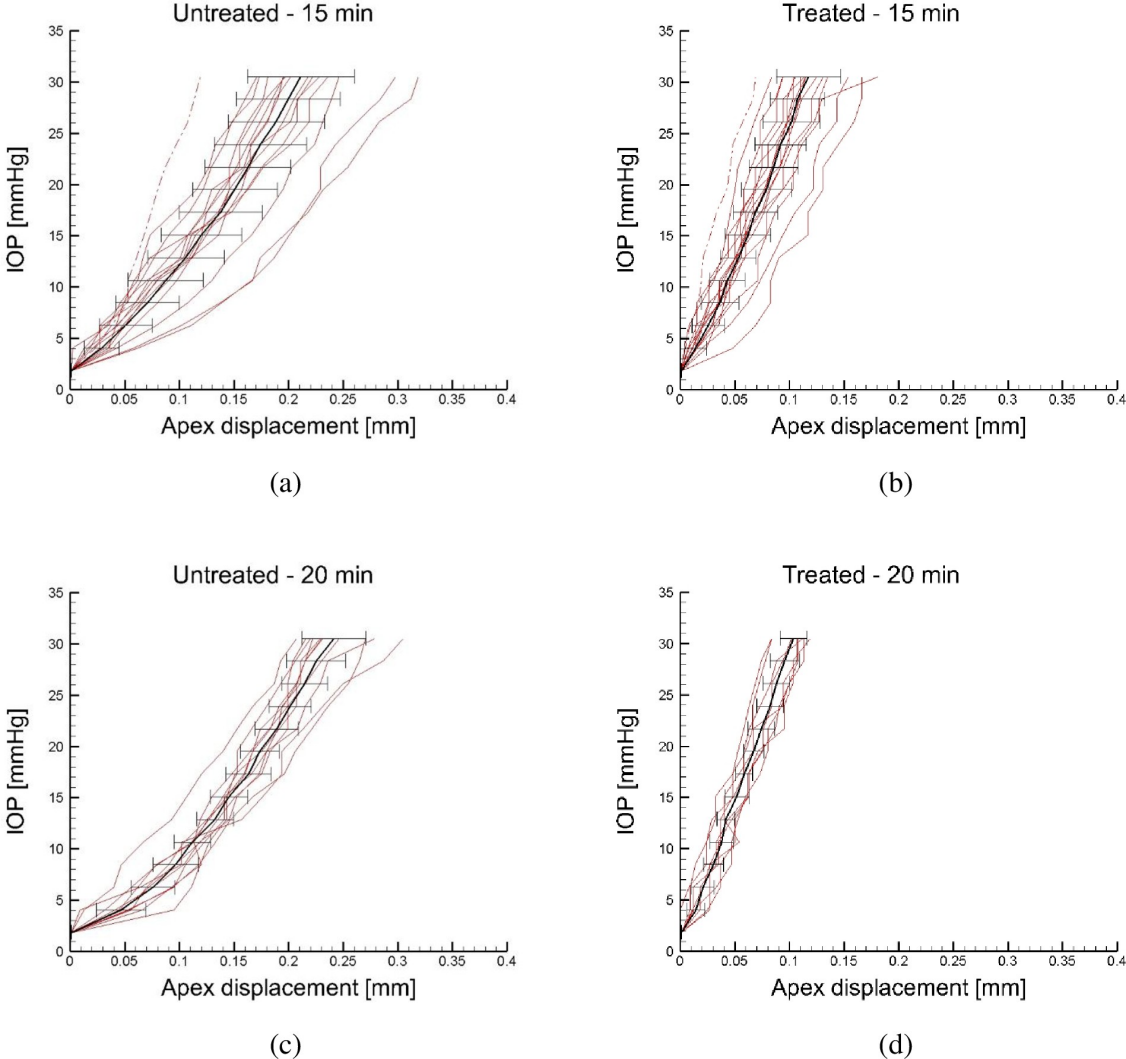
Inflation tests for the cornea groups irradiated for longer times, 15 and 20 minutes, respectively. IOP versus apex displacement: comparison between the untreated corneas and the treated corneas. Average values and error bars are also reported.

Figs 5A–5F show, in terms of global IOP versus apex displacement plots of treated corneas, the results of all the inflation tests for the shorter irradiation times (2.5, 5, and 10 min irradiation protocols), together with the average and the error bars. The curves still show a slope increment around 10–16 mmHg IOP. Fig 6A–6D show, in terms of global IOP versus apex displacement plots of treated corneas, the results of the inflation tests for longer irradiation times (15 and 20 min irradiation protocols), together with the average and the error bars. These two curves show a less marked change in the slope. Interestingly, the slope of the curves at 30 mmHg appears to be the same for all the groups of treated corneas. Figs 5 and 6 demonstrate that the effect of CXL is to reduce the dispersion of the mechanical behavior around the average value, with an ostensible reduction of the standard deviation. No stress versus strain curves were extracted for the treated corneas, because the loss of the tissue homogeneity across the thickness invalidates the significance of the curves.

**Fig 7:**
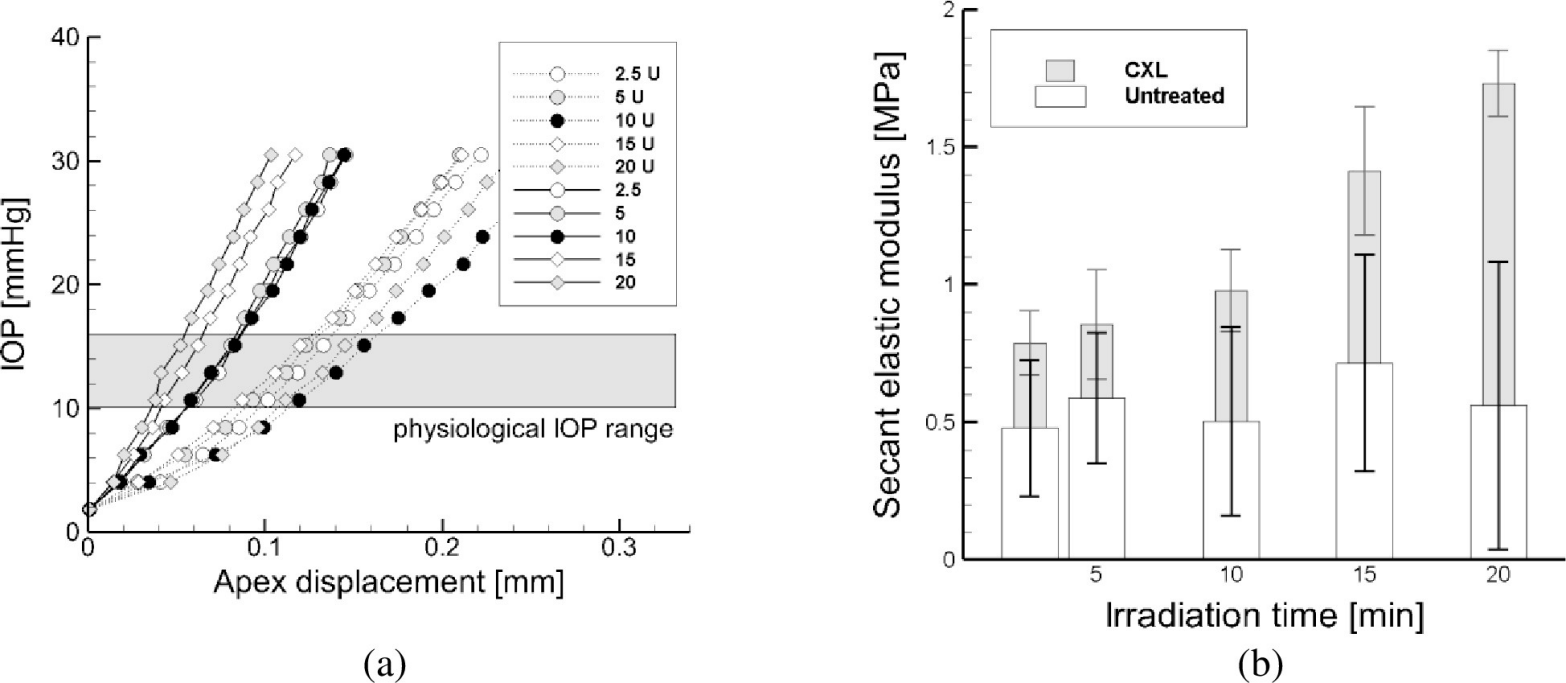
(a) Average pressure versus apex displacement curves. Comparison between the average untreated corneas (dotted lines) and the post CXL ones (solid lines), revealing a generalized stiffening. Corneas treated with shorter irradiation time protocol still show a change of stiffening around the physiological IOP, unlike the ones treated with longer irradiation time protocol, which show a rather linear behavior. Interestingly, in the treated corneas the slope at 30 mmHg is practically the same. (b) Average untreated E_b_ and post-CXL E_a_ cornea equivalent secant elastic modulus as obtained from the experiments.

In a synthetic and comparative plot, Fig 7A compares the average curves, without error bars, of the untreated and treated corneas of the five groups, to better visualize the difference between the effects of shorter and longer irradiation times protocols.

Fig 7B shows the average secant elastic modulus, obtained by averaging the values of each experimental curve at the physiological pressure, and grouped in untreated and post-CXL tests for each protocol. Average data and standard deviations are listed in Table 3. The U-Mann Whitney test was applied to each group of corneas, and showed a statistical difference (p < 0.05) between the corneal stiffness before and after CXL treatment, Table 4.

**Table 3:** Average values and standard deviation of the equivalent secant modulus obtained from the experimental data through the linearized shell theory.

Time	Secant Elastic Modulus [MPa]		
	Untreated	After CXL	T.Test
**2.5 min**	0.480 ± 0.247	0.788 ± 0.116	4.96E-04
**5 min**	0.590 ± 0.237	0.855 ± 0.200	1.05E-04
**10 min**	0.505 ± 0.342	0.978 ± 0.150	1.09E-04
**15 min**	0.716 ± 0.394	1.413 ± 0.234	9.12E-08
**20 min**	0.562 ± 0.522	1.732 ± 0.119	3.29E-04

**Table 4:** Statistical differences between the groups of tests.

Experiments	Test U-Mann Whitney
2.5 min—5 min	**0.299828**
2.5 min—10 min	**0.113203**
2.5 min—15 min	0.000269
2.5 min—20 min	0.000500
5 min—10 min	**0.105562**
5 min—15 min	0.001107
5 min—20 min	0.003093
10 min—15 min	0.015509
10 min—20 min	**0.143114**
15 min—20 min	**0.179394**

A statistical difference (p < 0.05) was observed between the 2.5 min irradiation protocol and both 15 and 10 min protocols, between 5 min and both 15 and 20 min protocols, and between 10 and 15 min protocols. No statistical differences (p > 0.05) were detected between the 2.5, 5 and 10 min protocols.

## 4. Discussion

The mechanical behavior of the porcine cornea resembles closely the behavior of the human cornea, therefore the results of the present experimental campaign can be used to understand the response of human corneas to CXL procedure. Images of the deforming corneas taken at different pressures provide a large database of results that have been employed for further numerical elaborations [22]. Nevertheless, synthetic plots of global parameters, such as the applied pressure and the apex displacement, provide an immediate representation of the mechanical behavior and are easily interpreted, understood, and analyzed statistically; furthermore, they are comparable to other studies often synthetized in terms of inflation curves.

Unlike in all previous studies [15, 23–25], inflation tests reported here were conducted on the same cornea specimens before and after CXL treatment. Inflation tests conducted before the CXL treatment confirmed the non-linear mechanical behavior with large data dispersion typical of untreated porcine corneas [20] and other soft biological tissues. In particular, around the physiological IOP the cornea attains a significant increment of the stiffness, marked by the change of the slope in Fig 4A, that can be attributed to the straightening of the collagen fibrils [21], confirming the trend observed in previous studies [20, 23]. The same behavior, characterized by a higher stiffness, is observed in the post-CXL corneas treated with a shorter irradiation time, Fig 5 (right column).

A direct comparison of the untreated and post-CXL corneas for each protocol is shown in Figs 5 and 6: plolshts on the left describe the untreated cornea behavior and plots on the right show the post-CXL cornea behavior. Pre-treatment tests are characterized by a wide dispersion and large standard deviation, typical of biological tissues. Contrariwise, post-CXL tests are characterized by a moderate dispersion around the average. The most evident effects of CXL on porcine corneas is to increase the stiffness over all the range of the investigated pressures (up to 30 mmHg), and to reduce the biological variability of the mechanical response, with a more marked effect for long exposure times. Compared to the control corneas, corneas treated for short exposures reveal a different stiffness at low and high IOP values (Fig 5), while for long exposures the bilinear behavior is lost (Fig 6). This effect can be more appreciated in Fig 7A. A prolonged irradiation time seems to be characterized by a generalized stiffening that includes also the low strain range, suggesting that all the potentially available crosslinks have been formed, and the occurrence of a sort of saturation of the process for which no longer exposure times are necessary [16].

For each cornea, the IOP versus apex displacement curves have been combined with the geometrical data (measured from the images with a specific software) to compute the equivalent secant elastic modulus. The equivalent secant elastic modulus is defined as the slope of the straight line connecting a point of the uniaxial stress-strain curve with the origin, and it has been obtained through the linearized shell theory, Eq (3). The adjective “equivalent” is mandatory: the shear modulus (the only modulus that has been tested at different depths) of the human cornea is not uniform across the thickness, showing larger values on the anterior third and reduced values in the posterior side. A similar configuration has been observed also in porcine corneas, with a reduction of the modulus of about one third [26].

The values of the equivalent modulus obtained from each test have been averaged in correspondence of a IOP of 15 mmHg, assumed as physiological value, see Fig 7B and Table 3. Furthermore, the shell theory supplied the equivalent biaxial stress-strain relationship for untreated corneas, Fig 4B, which confirms previous results [20, 21].

The experimental data allow to compare the average IOP versus apex displacement curves for untreated and post-CXL groups of corneas, see Fig 7A. Note that the five groups manifest a different behavior before the CXL, possibly because each group was built on a different delivery from the abattoir. Specifically, corneas undergoing the maximum exposure times (15 and 20 min, respectively) were characterized by a softer behavior in the untreated tests. The strong increment of stiffness observed in the post-CXL tests for these two cases suggests that a longer exposure caused a stronger effect on the corneal stiffness, in percentage more relevant than the one obtained in the short exposure cases. Again, the observed increase of stiffness with the exposure time (and therefore with the energy dose) is consistent with previous results [25, 15].

A statistical analysis with U-Mann Whitney test showed that low dose protocols (2.5 min, 5 min, and 10 min) have a statistical difference with high dose protocols (15 and 20 min) and no statistical difference among themselves, with the exception of the 10 min exposure time versus the 15 min case. The variability of the stiffening of the cornea with the irradiation dose requires to be investigated. The Bunsen-Roscoe law, stating that a photochemical reaction should remain constant if the total energy delivered is constant, is considered as a reference for CXL applications. The study [9] has demonstrated that the Bunsen-Roscoe law does not fully apply in the case of ACXL, because high-UV irradiance with short irradiation times reduces significantly the stromal oxygen diffusion capacity and the overall treatment efficiency in biomechanical terms.

The tests illustrated here have been conducted as variants of the A-CXL protocol (9 mW/cm^2^ irradiance for 10 min); for obvious reasons, the in-vitro investigation has been limited to the immediate post treatment with no further follow-up. The results of the present study encourage the use of A-CXL procedures with high energy doses, that, with respect to the Dresden protocol, reduce the CXL intervention time and are better tolerated by the patients. Recent clinical studies have shown that, during the first year follow-up in the stabilization of keratoconus, the A-CXL protocol with 9 mW/cm^2^ power and 5.4 J/cm^2^ dose provided outcomes comparable with the conventional 3mW/cm2 Dresden protocol [17, 18]. The two protocols seem to lead to the same level of crosslink formation. One can be concerned that the high intensity of the radiation may induce tissue damage. Interestingly, clinical observations have shown that A-CXL does not induce tissue damage such as the alteration of the extracellular matrix and more general wound related complications. In humans treated with high energy doses, long term in-vivo investigations by means of scanning laser confocal microscopy and corneal optical coherence tomography have shown time-dependent tissue modifications, without deterioration of corneal matrix [27]. Furthermore, in patients treated with high doses, in vivo thermography analysis showed no matrix injuries or heat-dependent corneal collagen denaturation in A-CXL [28].

The results obtained here show that a small dose is able to provide an appreciable increment of the corneal stiffness, although the longer the exposure time, the stronger the effect. This observation can be useful in the case of patients having a thin (inferior to 400 μm thickness) cornea, for which a reduction of irradiation times in the A-CXL protocol is considered to be opportune [29].

Although previous studies concerning the evaluation of the post-CXL cornea stiffness can be found in the literature, the present investigation differs from previous works for two main reasons. First, the study provides quantitative estimates of an elasticity parameter, the secant elastic modulus at physiological IOP, which possesses a precise meaning in the mechanics of soft tissues, and the comparison between treated and untreated corneas has been done on this parameter. Second, previous studies estimated the performance of CXL treatments by comparing different sets of corneas, while in the present study the same corneas underwent inflation tests before and after the CXL treatment. The relevance of using the same set of corneas for the two tests can be appreciated by observing that different sets of untreated corneas have shown strong discrepancies in the mechanical behavior, see Fig 7A and Table 5.

**Table 5:** Comparison of the experimental elastic modulus obtained in previous works concerning animal and human CXL procedures.

Reference	Cornea	Modulus			Treatment	
		Type	Natural [MPa]	Treated [MPa]	Dose [J/cm^2^]	Power [mW/cm^2^]
Wollensak et al, 2003	Porcine	Tangent (4–8%), strip	0.8–2.6	1.4–5.3	5.4	3
Wollensak et al, 2003	Human	Tangent (4–8%), strip	0.8–2.2	3.0–11.8	5.4	3
Hammer el al, 2014	Porcine	Tangent (10%), strip	11.5	12.9–16	5.4	3–18
Bao et al, 2018	Rabbit	Shear, inflation	0.01	0.03	5.4	3–90
Zhou et al, 2019	Rabbit	Average, elastography	0.09	0.14	5.4	3–18
Present work, 2020	Porcine	Secant (5%), inflation	0.55	0.8–1.7	1.5–10.8	9

Results obtained here can be used to gain awareness on the response to CXL of human corneas as well, although porcine corneas are much thicker than human corneas. In the mathematical expression given by Eq (3), however, the thickness does not represent an issue, since it is a parameter. Furthermore, on the basis of our previous experience with the interpretation of experimental data on porcine eyes [30, 31] the porcine stroma shows mechanical properties very similar to the ones of the human stroma.

The values of the secant elastic moduli here evaluated deepen the knowledge on corneal mechanical properties, with respect to similar studies presented by other authors.

In one of the pioneering works on CXL [8] both human and porcine corneas were tested under uniaxial loading on excised corneal strips. Values of the tangent elastic modulus for the untreated porcine corneas, strained at 4% to 8%, were in the range 0.8 to 2.6 MPa, and for treated corneas 1.4 to 5.3 MPa, values that compare well with the present study, where secant moduli are computed. The Bunsen-Roscoe law has been investigated in [13] by means of tests on porcine corneas using the same dose but different irradiation powers and times. The mechanical properties were tested under uniaxial loading on excised corneal strips, providing a tangent elastic modulus of about 11.5 MPa for untreated corneas and in the range 12.9–16 for CXL corneas, much higher than the present values. The difference can be easily explained because the measurements were taken at a strain (10%) much larger than the strain activated in physiological conditions (4%). Also the numerical analyses on inflation tests conducted on rabbit corneas by [15] reported an increase in the shear modulus of the treated corneas with respect to untreated. The numerical analyses estimated a 0.01 MPa shear modulus for the group of untreated corneas, and up to 0.03 MPa for the groups of treated corneas. In a subsequent work [32], using in-vivo elastography, the elastic modulus at the physiological IOP was measured as 0.09 MPa for untreated corneas and 0.14 MPa for CXL corneas irradiated at different powers.

The results obtained in this study are able to describe the link between the irradiation energy and the secant modulus of the porcine cornea in the short term. Clearly, in order to complete the analysis and to gain greater awareness of the results in the long term, in vivo animal testing is needed.

A more detailed description of the corneal mechanical behavior in response to CXL can be obtained by adapting numerical models such as [33], and implementing a procedure that models the formation of the cross-links in biochemical terms in order to evaluate the effects of irradiation in a more precise way, in terms of duration of exposure, quantity of energy and power emitted. Numerical or theoretical procedures can be used, then, to predict the distribution of the reinforcement across the thickness, as observed in the clinical practice. These aspects are outside the scope of this research but could constitute a future line of study.
